# Challenges in Epidemiological and Statistical Evaluations of Effect Modifiers and Confounders

**DOI:** 10.3389/fpubh.2014.00277

**Published:** 2014-12-10

**Authors:** Su Yon Jung

**Affiliations:** ^1^Translational Sciences Section, School of Nursing, University of California Los Angeles, Los Angeles, CA, USA

**Keywords:** interactions, effect modifiers, confounders, mediators, effect sizes

In multiple adjusted regression models, researchers sometimes do not know when the interaction occurs and how to interpret the exposure effect estimate while adjusting for the interaction term, resulting in a misinterpretation of the results; this issue has been raised in previous epidemiologic studies. In addition, when the positions of exposure and outcome are switched in the multiple regression, interpreting covariates is challenging.

Here, we present the epidemiological and statistical challenges in evaluating the effect modifier and confounding factor.

## When Does the Interaction Occur?

As an example to illustrate aspects of the evaluation of interaction, we describe below. In our example, we want to determine whether radon exposure (the third factor) is an effect modifier in the relationship between smoking (exposure) and lung cancer (outcome). For a dichotomous potential modifier [radon exposure (yes/no)], the interaction occurs when the effect of the exposure (smoking) on the outcome (lung cancer) is not homogenous in strata formed by a third variable (radon exposure) (1–3). The effect can be measured either by the attributable risk (in the additive model) or by a relative risk (in the multiplicative model); both models share the same conceptual basis for evaluating the interaction (1, 2).

To measure the effect of the interaction between smoking and lung cancer, we set the simple regression model and added a third variable, radon exposure, along with an interaction term of radon exposure with smoking (i.e., radon*smoking).

The regression model is shown below.

$$\begin{array}{llll} & Y\;\left(\text{lung}\;\text{cancer}\right)\; & & \;\\ & \;=B_{\text{s}}X_{\text{smoking}}+B_{\text{r}}X_{\text{radon}\;\text{exposure}}\; & & \;\\ & \;+B_{\text{sr}}X_{\text{smoking*radon}\;\text{exposure}},\; & & \; \end{array}$$

where *B*_s_ is the effect of X_smoking_, *B*_r_ is the effect of X_radon exposure_, and *B*_sr_ is the effect of X_smoking*radon exposure._

The most important issue in this model for determining whether or not radon exposure is an effect modifier is to interpret the effect size (i.e., *B*_sr_); that is, rather than focusing on only the “*p*-value” of the effect size, researchers should concentrate on the magnitude of the interaction term. In addition, when outcome variables are continuous and the levels of a continuous variable are small (e.g., 0.01, 0.02, and 0.03), the effect size of the interaction can be small resulting in a small slope in a graph composed of an *x*-axis for exposure and a *y*-axis for outcome. This occurs as a result of the tiny interval between outcome variable numerals; it does not suggest that there is no interaction. In this case, we can graphically test the interaction by plotting the means of the outcome variables for each category of exposure according to the strata defined by the effect modifier. Non-parallel lines suggest the presence of interaction. In both statistical and graphical evaluations, we always focus on the effect size of the interaction term rather than only its *p*-value.

## In the Regression Model That Includes Effect Modification, Why Does the Effect of Exposure Variable (*B*_s_) Not Represent the Relationship between Exposure and Outcome?

When the effect modifier (radon exposure) is determined to evaluate its interaction with exposure (smoking) and outcome (lung cancer), it should never be treated as a confounder in the analysis; the interaction effect (*B*_sr_) should be considered. In addition, the effect of smoking (*B*_s_) in a regression analysis in which the effect modification (*B*_sr_) is adjusted does not solely indicate the association between exposure and outcome, which has interacted with the effect modifier (4). The regression model shown below accounts for the effect modification:

$$\begin{array}{llll} & Y\;\left(\text{lung}\;\text{cancer}\right)\; & & \;\\ & \;=B_{\text{s}}X_{\text{smoking}}+B_{\text{r}}X_{\text{radon}\;\text{exposure}}\; & & \;\\ & \;+B_{\text{sr}}X_{\text{smoking*radon}\;\text{exposure}}.\; & & \; \end{array}$$

When the radon exposure status is no, the effect of smoking is *B*_s._

When the radon exposure status is yes, the effect of smoking is *B*_s_ + *B*_sr._

The above two cases of the dichotomous effect modifier indicate that the effect of smoking is not *B*_s_ only and that it depends on the effect of the interaction (*B*_sr_). Thus, when there is an interaction between smoking and radon exposure in lung cancer (i.e., *B*_sr_ has a noticeable effect), the effect of smoking must be measured in separate analyses, stratified by radon exposure status, to produce the effect of smoking within the radon exposure groups (*B*_s_; *B*_s_ + *B*_sr_).

## Interpreting Covariates (Confounders or Mediators) in the Regression Model Where the Positions between Exposure (X) and Outcome (Y) are Switched is Challenging

**Figure 1:**
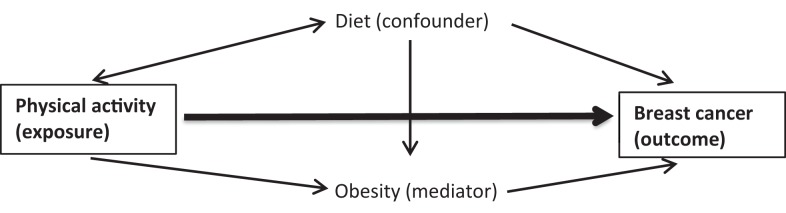
**Diagram describing the relationships between the three Xs and breast cancer outcome**.

Researchers sometimes face the situation where the positions between exposure and outcome are switched. For example, in a multiple regression model (see the below formula and Figure 1) of the relationship between physical activity (exposure) and breast cancer (outcome), we evaluate the effect of physical activity on breast cancer risk after accounting for the third factors such as diet (as a confounder) and obesity (as a mediator), as shown below:

$$\begin{array}{llll} & Y\;\left(\text{breast cancer: outcome}\right)\; & & \;\\ & \;=B_{\text{p}}X_{\text{physical activity}}\left(\text{exposure}\right)\; & & \;\\ & \;+B_{\text{d}}X_{\text{diet}}\left(\text{confounder}\right)\; & & \;\\ & \;+B_{\text{o}}X_{\text{obesity}}\left(\text{mediator}\right).\; & & \; \end{array}$$

The diagram in Figure 1 depicts the relationships between the three Xs and breast cancer outcome.

If researchers want to switch the positions between exposure and outcome, the following formula appears:

$$\begin{array}{llll} & Y\;\left(\text{physical activity: outcome}\right)\; & & \;\\ & \;=B_{\text{b}}X_{\text{breast cancer}}\left(\text{exposure}\right)\; & & \;\\ & \;\;+B_{\text{d}}X_{\text{diet}}\left({\text{1}}^{\text{st}}\;\text{of the third variables}\right)\; & & \;\\ & \;\;+B_{\text{o}}X_{\text{obesity}}\left(2^{\text{nd}}\text{ of the third variables}\right)\;.\; & & \; \end{array}$$

The new model does not appear to be mathematically problematic. However, the diet variable (*X*_diet_) is no longer a confounder because *X*_diet_ has no causal direction to physical activity outcome. Additionally, the causal relation between exposure and outcome is revered. For the same reason, the obesity variable (*X*_obesity_) is not a mediator (i.e., obesity 
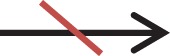
 physical activity; breast cancer 
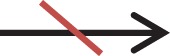
 physical activity). Therefore, in this new formula, *B*_b_ does not reflect the effect of breast cancer adjusted for the confounder and mediator. Furthermore, when researchers set a multiple regression model, they should first understand whether the third variables are included as confounders, mediators, or effect modifiers.

Overall, when using the formula for switching between exposure and outcome and adding third variables without considering their technical roles in a given multiple regression analysis, the results cannot be interpreted correctly to determine the effect of the exposure after adjusting for third variables.

In conclusion, previous epidemiological studies to evaluate effect modification using the multiple regression model may have overlooked an important issue in interpreting the effect modifier estimate by focusing on only the *p*-value rather than the effect size. Additionally, they may have misinterpreted the exposure effect in the regression analysis adjusted for the effect modification. Moreover, when the positions are switched between exposure and outcome in the multiple regression, the third variables in the formula do not have the same epidemiological roles as those in the formula prior to the switch; they need to be considered carefully to determine their roles as confounders, mediators, or effect modifiers.

### Conflict of Interest Statement

The author declares that the research was conducted in the absence of any commercial or financial relationships that could be construed as a potential conflict of interest.
